# Confounding by indication and exposure misclassification may undermine corticosteroid effect estimates in ICU patients with alcohol-related hepatitis

**DOI:** 10.1186/s13613-025-01486-4

**Published:** 2025-05-19

**Authors:** Dongxu Mao, Min Li

**Affiliations:** The Affiliated Jiangning Hospital of Nanjing Medical University, Nanjing, 211166 China

To the Editor,

We read with great interest the recent multicenter study by Gasperment et al. [1], which assessed clinical features and mortality risk in patients with alcohol-related hepatitis (ARH) admitted to intensive care units (ICUs), including an evaluation of corticosteroid treatment effects using overlap weighting. While we commend the authors for assembling a unique and clinically important cohort, we urge caution in interpreting their conclusion that corticosteroids offered no survival benefit in ICU patients.

Patients treated with corticosteroids were clearly less severe at baseline, with lower MELD and SOFA scores, lower lactate levels, and lower rates of sepsis. Although overlap weighting is a sophisticated adjustment method, it cannot control for unmeasured confounding, particularly when treatment assignment is driven by clinician judgment, as in ICU settings. This is a textbook example of confounding by indication, where clinicians may withhold steroids from the sickest patients due to perceived futility or infection risk. Such confounding persists despite statistical adjustment [2]. In ICU environments, treatment choices are seldom random and often reflect nuanced clinical decisions based on undocumented impressions of patient frailty, likelihood of response, or risk of complications. Propensity score models, including overlap weighting, rely on the assumption of no unmeasured confounding (strong ignorability), which is rarely satisfied in such settings [3].

Moreover, the inclusion of patients who received hydrocortisone for septic shock under the corticosteroid group introduces exposure misclassification, conflating distinct treatment indications. This non-differential misclassification likely biased estimates toward the null. Treating glucocorticoids as a uniform exposure obscures important differences between indication-specific prescriptions (e.g., immunomodulation in ARH vs. stress-dose steroids in septic shock). Epidemiologic theory and pharmacoepidemiologic standards caution against this practice [4].

In addition, the absence of sensitivity analyses—such as E-values to quantify the robustness of the null findings—limits confidence in the reported results. E-values help determine how strongly an unmeasured confounder would need to associate with both treatment and outcome to explain away observed associations—or lack thereof. Without these analyses, the apparent lack of effect may reflect Type II error or residual bias.

Furthermore, the study did not emulate a target trial—a framework increasingly advocated for causal inference in observational studies. Without clear definitions of eligibility, treatment initiation, and time-zero alignment, the risk of immortal time bias and reverse causation increases [5]. Corticosteroid administration after ICU admission may reflect survival bias, where only patients who lived long enough or stabilized were eligible for treatment, again underestimating the true effect.

Despite these concerns, this study is an important contribution and highlights the need for more methodologically rigorous investigations into the management of ARH in critically ill populations. Future research should consider employing marginal structural models or instrumental variable approaches and ensure clear separation of treatment groups based on indication and timing. These tools would enhance the credibility of treatment effect estimates and better inform clinical decisions in this high-risk group.
